# Chromodomain mutation in *S*. *pombe* Kat5/Mst1 affects centromere dynamics and DNA repair

**DOI:** 10.1371/journal.pone.0300732

**Published:** 2024-04-25

**Authors:** Tingting Li, Ruben C. Petreaca, Susan L. Forsburg

**Affiliations:** Program in Molecular & Computational Biology, Department of Biological Sciences, University of Southern California, Los Angeles, CA, United States of America; Tulane University Health Sciences Center, UNITED STATES

## Abstract

KAT5 (*S*. *pombe* Mst1, human TIP60) is a MYST family histone acetyltransferase conserved from yeast to humans that is involved in multiple cellular activities. This family is characterized in part by containing a chromodomain, a motif associated with binding methylated histones. We show that a chromodomain mutation in the *S*. *pombe* Kat5, *mst1-W66R*, has defects in pericentromere silencing. *mst1-W66R* is sensitive to camptothecin (CPT) but only at an increased temperature of 36°C, although it is proficient for growth at this temperature. We also describe a de-silencing effect at the pericentromere by CPT that is independent of RNAi and methylation machinery. We also show that *mst1-W66R* disrupts recruitment of proteins to repair foci in response to camptothecin-induced DNA damage. Our data suggest a function of Mst1 chromodomain in centromere heterochromatin formation and a separate role in genome-wide damage repair in CPT.

## Introduction

Kat5 is a member of the MYST family histone acetyltransferases (HATs), and is an essential protein conserved across eukaryotes (rev. in [1, 2]). It is known as Mst1 in *S*. *pombe*, Esa1 in *S*. *cerevisiae*, and TIP60 in mammals (rev. in [3, 4]). In humans, TIP60 has also been identified as a tumor suppressor (rev. in [5, 6]) and as a therapeutic drug target (rev. in [7]). Kat5 functions as the catalytic subunit of a multi-protein complex called NuA4, and acetylates a number of different histone residues (rev. in [8]). NuA4 functions in coordination with the histone remodeling complex Swr1, which is responsible for incorporation of the histone variant H2A.Z into chromatin (rev. in [9]). Kat5 has pleiotropic functions in a wide variety of nucleic acid transactions, including transcriptional regulation, chromosome segregation and DNA double strand break (DSB) repair (rev. in [4]). In addition, some studies suggest that Kat5 may also acetylate non-histone proteins including ATM and the ssDNA binding protein RPA [10, 11].

In multiple organisms, Kat5 contributes to heterochromatin formation. For example, in mouse cells, TIP60 localizes at the pericentromeric region and is required for proper chromosome segregation [12]. In *S*. *pombe*, Mst1 acetylation of H3K4 contributes to the switch between two H3K9^me^ bound chromodomain proteins: the RITS complex component Chp1, and the HP1 protein orthologue Swi6 [13]. In addition, Mst1 also interacts with CENP-B-like protein Cbh1, suggesting a potential role at the centromere [14].

The N-terminus of Kat5 acetyltransferase itself contains the chromodomain, a motif that potentially binds to methylated lysines in histone proteins [15]. In human cells, TIP60 binds to histone H3K9^me^ at DSBs to facilitate repair [16]. Our previous work showed that *S*. *pombe* Mst1 binding near DSB depends on Clr4, the H3K9 methyltransferase [17]. Interestingly, the chromodomain motif is preserved in the budding yeast Esa1 protein, although H3K9 methylation is missing in that system.

Studies in various organisms have shown that Kat5 acetyltransferase contributes to DNA damage repair and Kat5 mutations have been identified in many cancers [18]. There is evidence that the recruitment of histone variant H2A.Z and its subsequent acetylation by Kat5 are essential early in the DNA damage response [19–22]. Kat5 is responsible for the turnover of H2A.Z via acetylation [23–27]. Additionally, Kat5 is responsible for the acetylation of the damage specific phosphorylated histone variant γ-H2A(X) that promotes its turnover [28–31]. NuA4 acetylation on ssDNA binding protein RPA also regulates resection at DSB [11]. This activity may be important to activate the homologous recombination pathway as opposed to other types of repair [32, 33]. Our previous study showed that Mst1 in *S*. *pombe* contributes to efficient resection at DSB through H2A.Z [17]. We further showed that the Clr4 H3K9 methyltransferase contributes to recruitment of Mst1 at a DSB. NuA4 in *S*. *pombe* has also been linked to proper repair of damage created by Camptothecin (CPT), a topoisomerase poison that causes replication fork breakage [34].

In this study, we investigated the contribution of the chromodomain motif to Mst1 activity in fission yeast. To facilitate comparisons across systems, we constructed an allele *mst1-W66R* in a highly conserved residue that corresponds to a mutation previously analyzed in *S*. *cerevisiae* [35]. We show that *mst1-W66R* is viable at all temperatures but is defective in heterochromatin silencing at the pericentromere domain. This mutant shows temperature-dependent defects in resistance to CPT treatment and unexpectedly, we find that CPT also destabilizes silencing. Our data further suggest that Mst1 is required for the global response to CPT and assembly of appropriate repair proteins.

## Results

### Construction and characterization of a chromodomain mutation in Mst1

All Kat5 family proteins have a chromodomain in the N-terminus, which is presumed to bind methylated histones (rev. in [15]). There is evidence from human cells that the chromodomain targets Kat5 to methylated H3K9 [16, 36, 37]. This histone modification is associated with heterochromatin assembly [13, 14] and has been linked to DSB repair (rev. in [38]). Our previous work demonstrated that fission yeast Mst1 contributes to resection and recruitment of repair proteins to DSBs, and also showed that this depends upon the H3K9 methyltransferase Clr4 [17], consistent with a canonical role for the Mst1 chromodomain.

Interestingly, the budding yeast *S*. *cerevisiae* lacks H3K9 methylation, although its Kat5 orthologue Esa1 preserves the conserved chromodomain. A mutation in the highly conserved residue in the chromodomain of *S*. *cerevisiae* Esa1 W66R causes impaired histone H4 acetylation and sensitivity to the genotoxin camptothecin [35].

**Fig 1:**
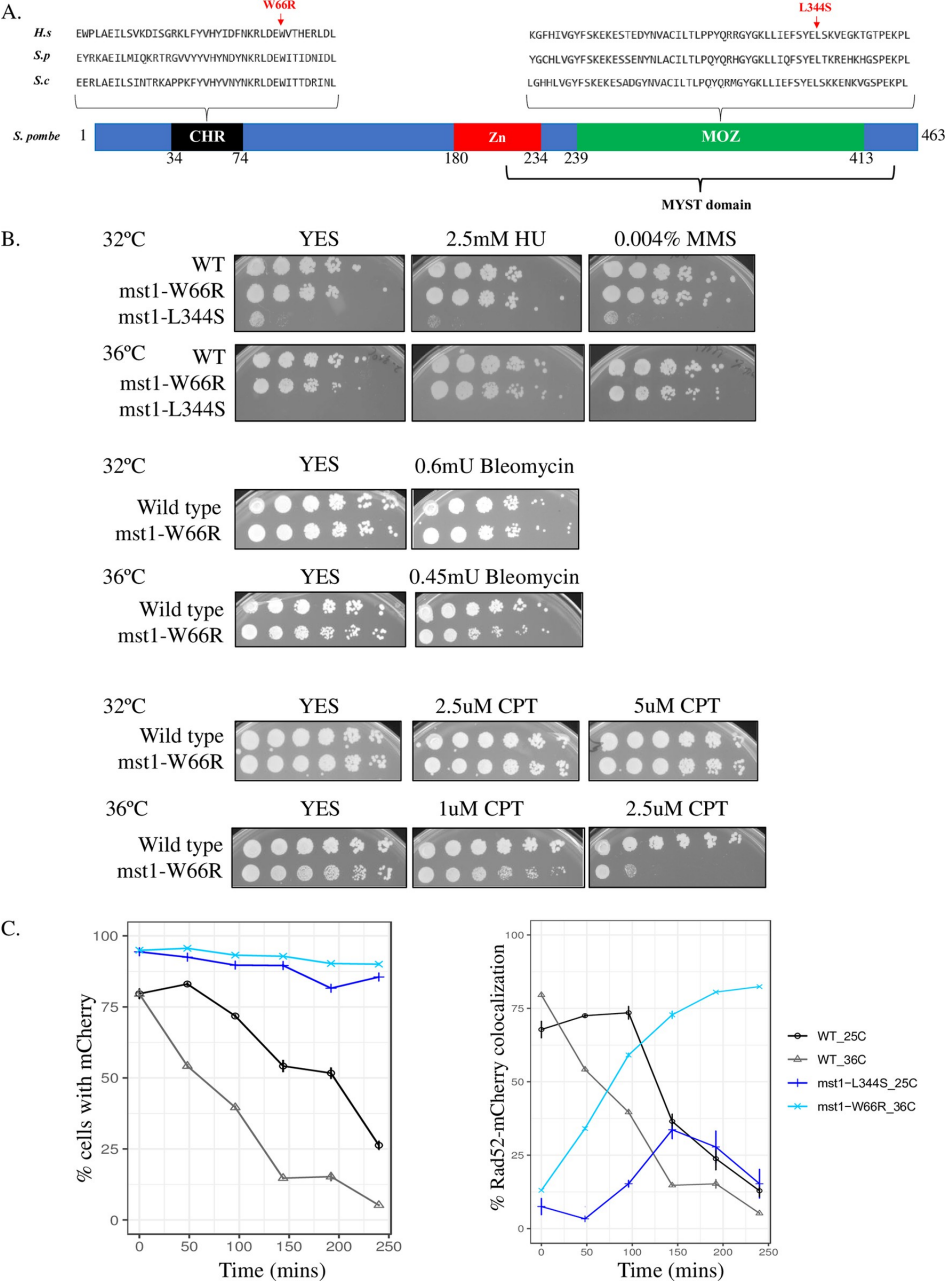
*mst1-W66R* allele characterization. **(**A) The alignment of *H*. *sapiens* TIP60, *S*. *cerevisiae* Esa1 and *S*. *pombe* Mst1. The mutation at W66 (*S*. *pombe* and *S*. *cerevisiae* coordinates) and L344 (*S*. *pombe* coordinates) are marked with the arrow. (B) The *mst1-W66R* shows sensitivity to CPT at 36°C. Cells were grown in YES media overnight at 32°C then 5X serial dilutions were spotted onto YES plates or YES plates containing HU, MMS, Bleomycin or CPT. Plates were incubated at 32°C or 36°C and photographed after 4 days. (C) **Left**: Percentage of cells with HO-induced mCherry foci over 240 minutes in wild type and *mst1-W66R* cells at 36°C. **Right**: Percentage of cells with colocalized Rad52-CFP and HO-induced mCherry foci over 240 minutes in *mst1-W66R* cells.

We constructed the equivalent mutation in *S*. *pombe* to facilitate comparison between a system that maintains H3K9 methylation, and one that lacks it: the allele *mst1-*W66R that corresponds to *S*. *cerevisiae esa1-W66R* [35] (Fig 1A). This residue is also conserved in all *S*. *pombe* chromodomain proteins (S1 Fig).

We compared the phenotypes of *mst1-W66R* to those of the *mst1-L344S*, a previously characterized temperature sensitive allele which has a number of defects even under permissive conditions [14, 17]. Unlike the *mst1-L344S* allele which harbors a mutation in the catalytic domain of the enzyme (Fig 1A), the *mst1-W66R* mutant is viable at all temperatures, with no evidence for temperature sensitivity (Fig 1B). Previously we showed that *mst1-L344S* at the permissive temperature is extremely sensitive to genotoxic stressors including the radiomimetic bleomycin, the alkylating agent methyl methanesulfonate (MMS), nucleotide starvation drug hydroxyurea (HU), and topoisomerase (Top1) inhibitor camptothecin (CPT) [14]. We investigated whether *mst1-W66R* shows any similar sensitivities. We observed that *mst1-W66R* was not sensitive to bleomycin, HU, or MMS at any temperature. Curiously, we observed sensitivity to CPT, but only at 36°C (Fig 1B). CPT captures Top1 complexes on the DNA (rev. in [39]) which is associated with increased double strand breaks not only during DNA replication [40], but in transcription as well [41]. The fact that both *mst1* alleles show sensitivity to CPT underscores the role of Mst1 in DNA double strand break repair.

Our previous work showed that loss of the H3K9 methyltransferase Clr4 leads to reduced localization of Mst1 and the HR protein Rad51 to DSBs, and delayed resection [17]. Therefore, we investigated whether the *W66R* chromodomain mutation impairs resection and recruitment of a DSB. Because *mst1-W66R* CPT sensitivity is temperature sensitive, we examined these dynamics at 36°C. As before, we used a strain containing an HO-inducible DSB adjacent to a lacO array that recruits mCherry-lacI, and CFP-tagged Rad52 [42]. The strain produces persistent DSBs [43]. Following break induction, the two ends are first resected, then repair factors including Rad52 are recruited to the break. Loss of the mCherry signal corresponds to resection while an increased CFP signal indicates that RAD52-CFP has been recruited to the break. We imaged both wild-type and *mst1-W66R* cells after break induction at 36°C (Fig 1C; see materials and methods). We compared this to our prior study using *mst1-L344S* at permissive temperature, in which we observed resection defects [17]. We find that, similarly to *mst1-L344S*, the cherry signal was maintained in *mst1-W66R*, suggesting a reduced efficiency of resection. However, localization of the Rad52 protein to the DSB increases over time in *mst1-W66R*, whereas it declines in *mst1-L344S*, suggesting a defect in Rad52-CFP recruitment for *mst1-L344S* but not *mst1-W66R*. Thus, *mst1-W66R* also has a DSB repair defect but does not simply phenocopy *mst1-L344S*.

### *mst1-W66R* does not affect DNA damage transcription response

Because Mst1 has a role in transcriptional regulation [44], we investigated whether the temperature-dependent phenotypes in *mst1-*W66R are linked to changes in transcription specifically of DNA damage repair genes. We performed mRNA-sequencing to compare the transcription profile of *mst1-W66R* cells at 36°C compared to wild type. Using a threshold of |logFC|>1.5 and p-value<0.05, we found only 25 genes were significantly up-regulated and 27 significantly were down-regulated in *mst1-W66R* compared to wild type and most of these differentially expressed genes were non-coding RNAs (S2A Fig and S2 Table).

Next, we examined the transcriptional response in CPT-treated *mst1-W66R*. We observed that 48 genes were significantly up-regulated and 128 were significantly down-regulated in CPT-treated *mst1-W66R* compared to untreated (S2B Fig and S2 Table). Of these, 29 up-regulated genes in *mst1-W66R* were also up-regulated in wild type with CPT, and 35 down-regulated genes in *mst1-W66R* were also down-regulated in wild type with CPT (S2 Table). Most of the genes that were differentially expressed in CPT-treated *mst1-W66R* compared to wild type are involved in metabolism. Overall, we found that metabolic gene sets were enriched in CPT-treated *mst1-W66R* compared to untreated from KEGG analysis (S2C Fig), while aminoacyl-tRNA biosynthesis was also found depleted in CPT-treated *mst1-W66R* (S2D Fig). GO analyses and GSEA analyses were also conducted but no gene sets passed the threshold. These data indicate that the disruption in the CPT response is not predominantly due to disruption of transcription of damage response genes, nor a generalized transcription defect.

### Temperature specific silencing defects in *mst1-W66R*

**Fig 2:**
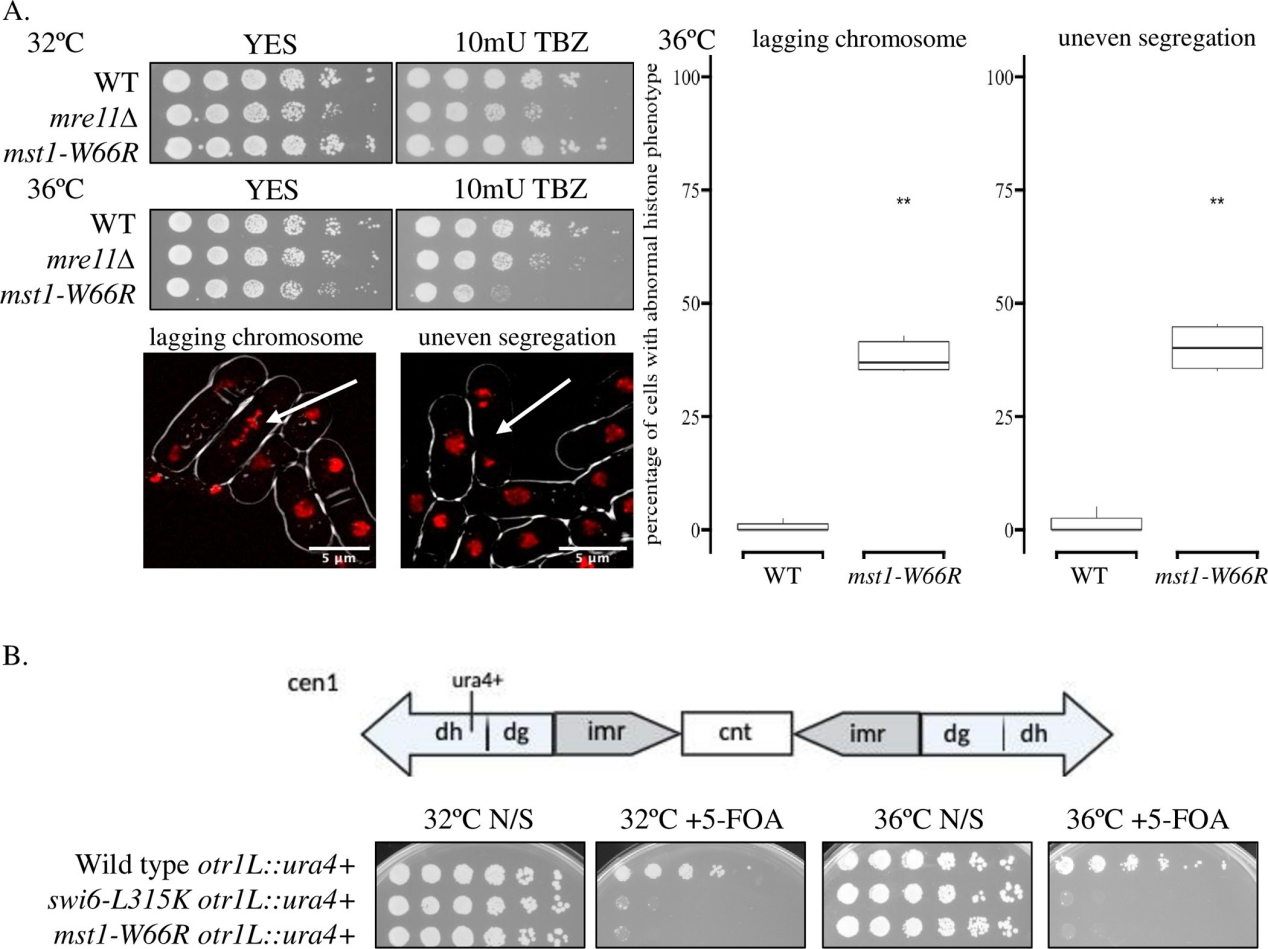
*mst1-W66R* showed temperature specific silencing defects. (A) **Left Top:** *mst1-W66R* growth in TBZ at 32°C and 36°C. *mre11Δ* as negative control. **Left Bottom:** Representatives of *mst1-W66R* cells with Hht1-mRFP showing lagging chromosome (left) and uneven segregation phenotype (right). **Right:** percentage of wild type and *mst1-W66R* cells with lagging chromosome or uneven segregation phenotypes at 36°C. (B) **Top:** Schematic of the *ura4*^*+*^ marker in the *cen1 dh* repeats. **Bottom:** expression of centromere Ura4 in different mutants at 32°C or 36°C was determined by sensitivity to 5-FOA. N/S, non-selective (Uracil+) medium.

Previous studies have suggested that in some systems, the conserved KAT5 chromodomain binds to H3K9^me^ histone [16, 35, 36]. In fission yeast, Clr4 methylates H3K9 that is essential for assembly of pericentric heterochromatin, which in turn contributes to faithful chromosome segregation (rev. in [45]). To investigate whether the Mst1 chromodomain contributes to heterochromatin function, we examined chromosome segregation in *mst1-W66R* at 32°C and at 36°C. We observed that cells showed modestly reduced colony size, increased rates of chromosome mis-segregation and lagging chromosomes, and increased thiabendazole (TBZ) sensitivity, most noticeably at 36°C (Fig 2A). This suggests an impairment in centromere function particularly at higher temperature.

We next examined whether *mst1-W66R* affects heterochromatin silencing at the centromere region, which depends on H3K9 methylation (rev. in [46]). Prior evidence shows that heterochromatin silencing is intrinsically temperature sensitive even in wild type cells [47, 48]. We utilized strains that contain a *ura4*^*+*^ reporter gene integrated at the outer repeat *otr1L(dh)* of centromere 1 (Fig 2B) [49]. Cells that fail to silence *ura4*^*+*^ properly can grow in non-selective media, but are inviable in media containing 5-fluoro-orotic acid (5-FOA). Wild type cells containing the reporter gene showed growth in both non-selective media and media containing 5-FOA, indicating that silencing at the centromere is intact. In contrast, the control strain *swi6-L315K* was killed by 5-FOA [50]. *mst1-W66R* showed similar silencing defects as *swi6-L315K* at both temperatures, suggesting that the Mst1 chromodomain contributes to the silencing of the outer pericentromeric regions.

### Temperature dependent CPT sensitivity among heterochromatin mutants

**Fig 3:**
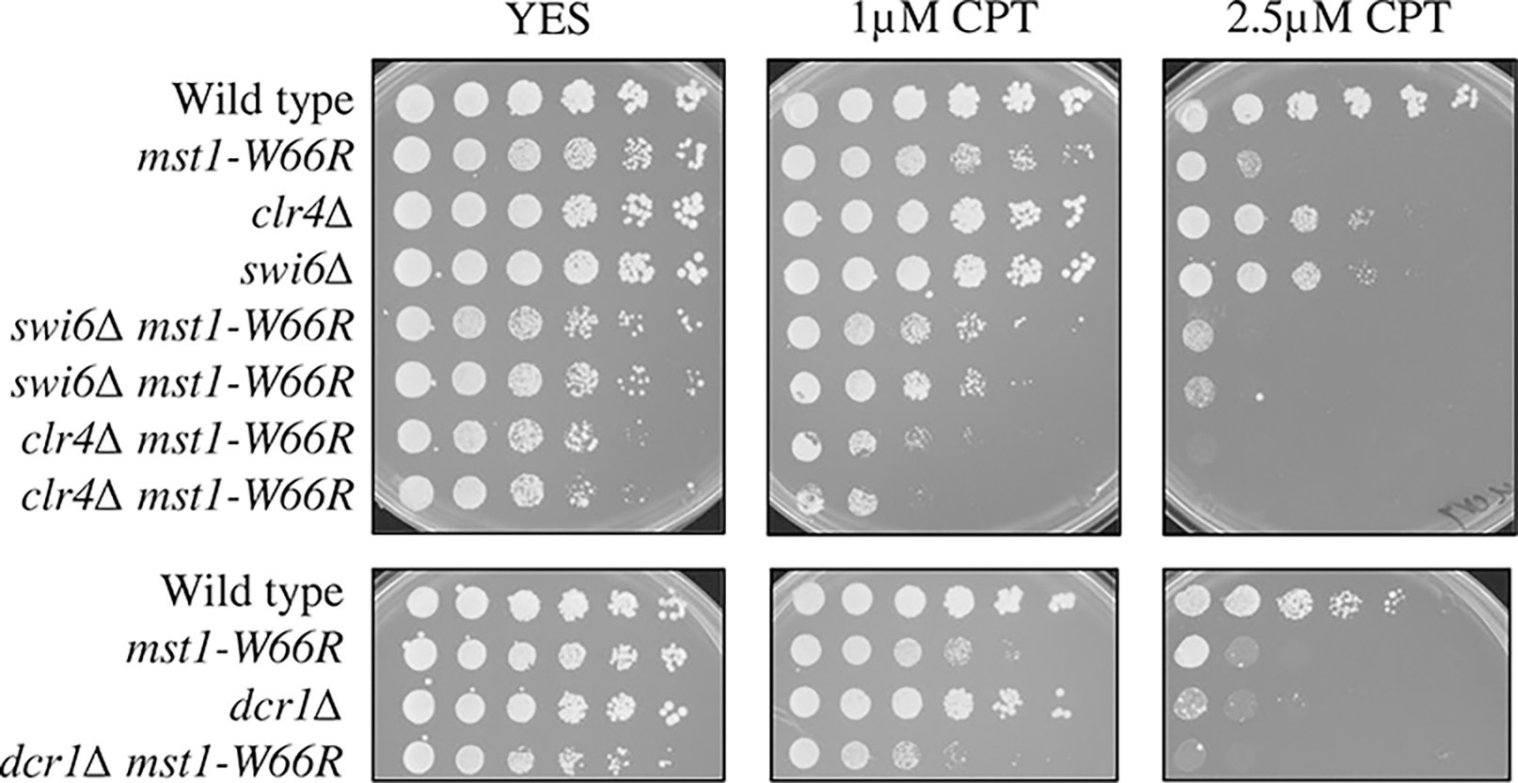
Heterochromatin mutants show sensitivity to CPT at 36°C. *clr4Δ*, *swi6Δ*, *dcr1Δ* and the double mutants with *mst1-W66R* showed sensitivity to CPT at 36°C. Cells were grown in YES media overnight at 32°C then 5X serial dilutions were spotted onto YES plates or YES plates containing CPT. Plates were incubated at 36°C and photographed after 4 days.

Since *mst1-W66R* showed a temperature-specific sensitivity in CPT as well as centromere silencing and increased chromosome mis-segregation at 36°C, we wondered whether these phenotypes were linked in some way. We investigated whether known heterochromatin mutants also show increased CPT sensitivity at higher temperature. We examined *clr4Δ*, disrupting the H3K9 methyltransferase that promotes heterochromatin formation [51, 52]; *swi6Δ*, disrupting the heterochromatin protein HP1 that binds to H3K9 methylated histone [51]; and *dcr1Δ*, which removes Dicer protein that promotes RNAi required for methylation [53]. We observed that the individual heterochromatin mutants all showed modest CPT sensitivity at higher temperature, but not as dramatic as *mst1-W66R* (Fig 3).

We constructed double mutants between these heterochromatin protein mutants and *mst1-W66R*. We found that *clr4Δ*, *swi6Δ* and *dcr1Δ* double mutants showed mild synthetic defects with *mst1-W66R* at 32°C which were increased at 36°C compared to *mst1-W66R* alone (Fig 3 and S3A Fig). This suggests that the *mst1-W66R* CPT phenotype is at least partly independent of H3K9^me^ by Clr4, and independent of its effects on heterochromatin.

**Fig 4:**
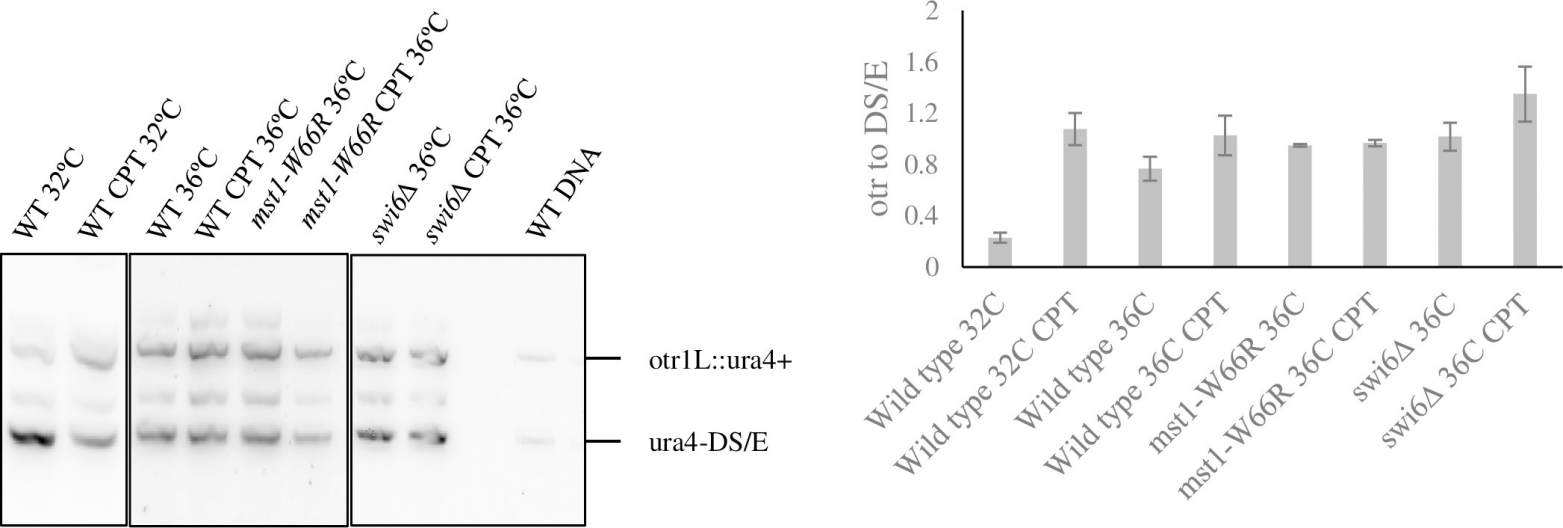
CPT de-silenced centromere but independent of Mst1. *ura4+* expression in *cen1L(dh*) measured using RT-PCR with or without four-hour 15μM CPT treatment at 32°C or 36°C. Signals were normalized to expression of the *ura4-DS/E* minigene at the normal (euchromatic) locus.

Next, we asked whether CPT treatment itself affects heterochromatin silencing. We performed reverse-transcriptase PCR on the *ura4*^*+*^ gene inserted at outer repeats of chromosome I (Fig 4, S1 Raw images). At 32°C, the signal at the outer repeat was only 0.2 times of that at the euchromatic locus, consistent with silencing. Intriguingly, after four-hour CPT treatment, the expression of *ura4*^*+*^ at the outer repeats increased to 0.8 times of that at the euchromatic locus, suggesting that CPT can partially disrupt silencing.

Previous studies showed that *S*. *pombe* cells lose pericentromere silencing at higher temperature, correlating with loss of RNAi activity as Dicer protein is exported out of nuclei [47, 48, 54]. Consistent with this, we observed that untreated wild type cells at 36° showed higher expression of outer repeats compared to 32°C. CPT treatment further increased this expression. Thus, increased temperature and CPT appear to have cumulative effects on silencing. Untreated *swi6Δ* and *mst1-W66R* strains show similar expression. Following 4hr CPT treatment, outer repeat expression in *swi6Δ* was slightly higher than that in wild type cells with CPT treatment. We observed that *mst1-W66R* cells at 36°C had increased expression from outer repeats compared to wild type cells at 36°C, but this was not further induced by CPT treatment.

### *Mst1-W66R* is dominant negative

We wondered whether *mst1-W66R* has a dominant negative effect. To address this hypothesis, we transformed *mst1-W66R* cells with an episomal plasmid expressing wild type Mst1 under the native promoter and Leu+ selective marker (S3B Fig). Even with the wild type Mst1 expressed in *mst1-W66R*, cells still showed sensitivity to CPT at 36°C, although not quite to the same extent. Increasing expression of *mst1+* on a plasmid with the *nmt1* promoter showed additional rescue (data not shown), consistent with a dominant negative phenotype.

### *mst1-W66R* is required to recruit repair proteins in CPT

**Fig 5:**
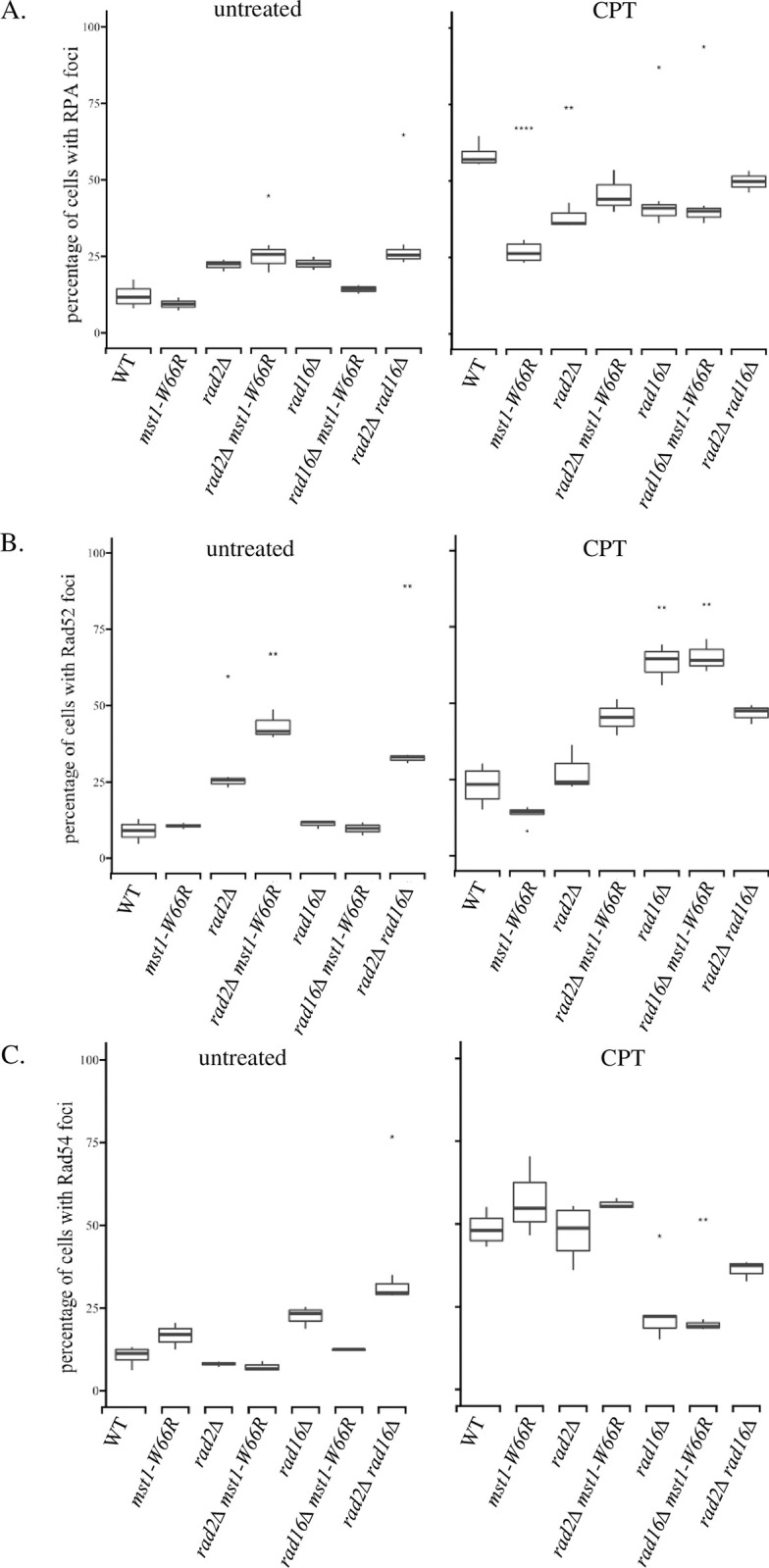
The *mst1-W66R* mutants affect recruitment of repair proteins at 36°C in CPT Percentage of cells with (A) RPA (Rad11) (B) Rad52 and (C) Rad54 foci in WT, *mst1-W66R*, *rad2Δ*, *rad2Δ mst1-W66R*, *rad16Δ*, *rad16Δ mst1-W66R* and *rad2Δ rad16Δ*. The bold line in each box represents median. Asterisks represent percentage of cells with foci is significantly different than that in WT (*: p<0.05, **: p<0.01, ****: p<5e-05 Mann-Whitney Test).

We speculated that the CPT sensitivity in *mst1-W66R* might be due to an additional function in repair of CPT-induced damage independent of the pericentromere silencing defect. We examined the response of *mst1-W66R* cells to CPT treatment by monitoring recruitment of repair proteins to characteristic repair foci, following four-hour treatment with CPT at 36°C. We first examined at the localization of RPA which binds single strand DNA (ssDNA) (Fig 5A). Untreated wild type and *mst1-W66R* cells have a similar low percentage of cells with RPA foci. However, after treatment with 5μg/mL CPT at 36°C, significantly more wild type cells (58.57%) have foci than *mst1-W66R* cells (26.76%) (S3 Table). The RPA foci in *mst1-W66R* were distributed throughout the nucleus, suggesting that RPA is not preferentially localized at the centromere, which is typically peripheral (S4A Fig).

We next investigated the recruitment of proteins involved in fork rescue and homologous recombination. Rad52 is associated with various recombination pathways including single-strand annealing (SSA), homologous recombination (HR), and synthesis dependent strand annealing (SDSA) (rev. in [55]) and is thought to facilitate RPA displacement [56] (Fig 5B). We observed that 10% of cells have Rad52 foci in both wild type and *mst1-W66R* background in untreated cells. Upon CPT treatment, there was a modest increase to about 25% of wild type cells showing Rad52 foci. However, in *mst1-W66R* treated with CPT, there was no increase of Rad52 signal, and the percentage of cells with Rad52 foci remained at about 10% (S3 Table).

We next investigated the role of Rad54, which functions downstream of Rad51 in homologous recombination and fork reversal [57, 58] (Fig 5C). In untreated cells, both wild type and *mst1-W66R* showed similar levels of Rad54-GFP foci, in around 10–15% of the cells. However, in contrast to our observations with RPA and Rad52, both wild type and *mst1-W66R* cells induced Rad54 foci to about 50% after CPT treatment.

Together, these imaging data show a distinct pattern of repair protein recruitment in *mst1-W66R*. To further understand these phenotypes and how they may reflect CPT sensitivity, we took a candidate approach to determine the involvement of known repair pathways in CPT response.

### Interactions with known repair pathways

**Fig 6:**
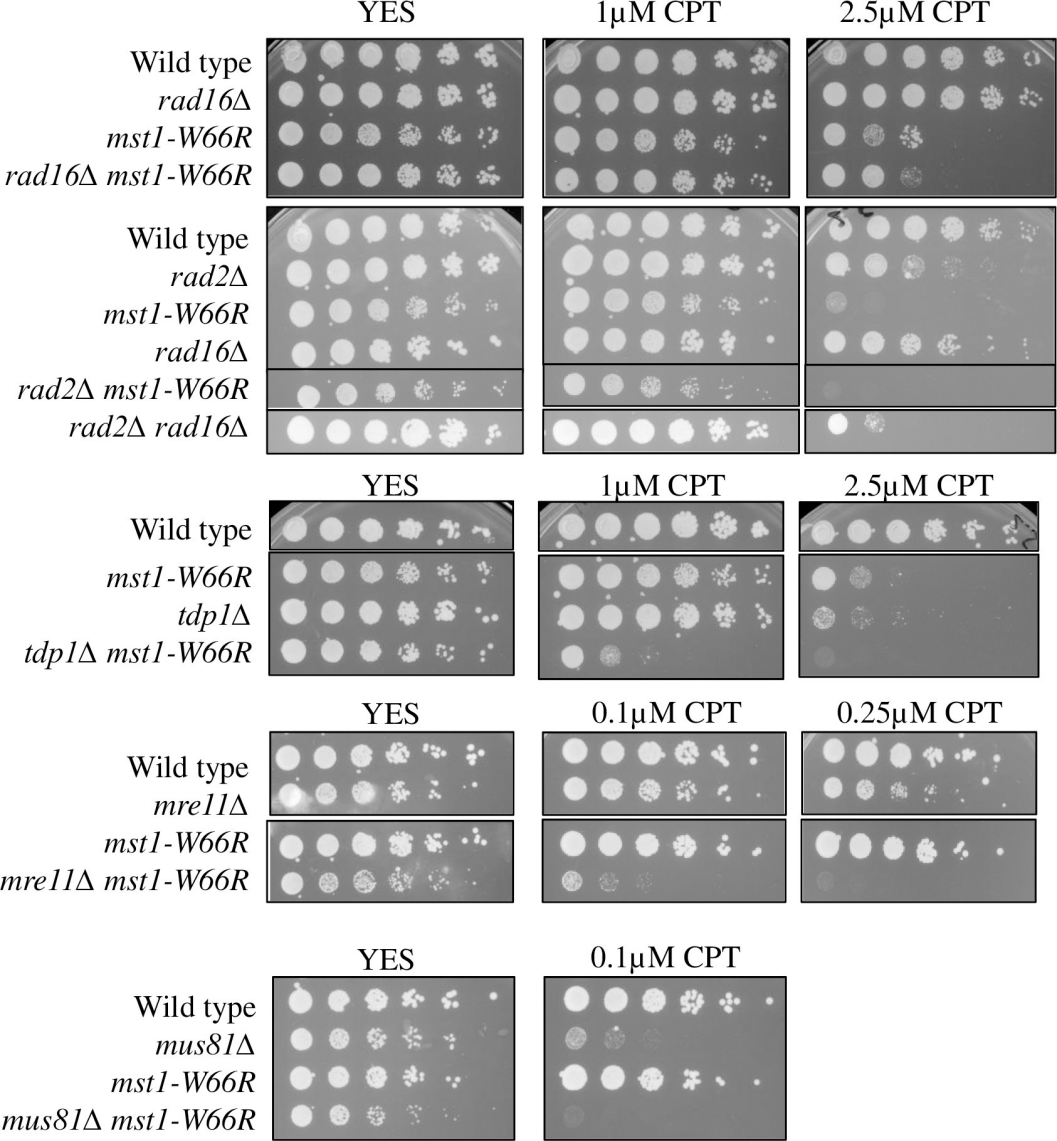
Genetic interaction between *mst1-W66R* and mutants involved in Top1cc removal. *rad16Δ*, *rad2Δ*, *tdp1Δ*, *mre11Δ*, *mus81Δ* and the double mutants with *mst1-W66R* sensitivity to CPT at 36°C. Cells were grown in YES media overnight at 32°C then 5X serial dilutions were spotted onto YES plates or YES plates containing CPT. Plates were incubated at 36°C and photographed after 4 days.

We investigated the genetic interaction between *mst1-W66R* and selected mutants that are known to facilitate CPT response, by constructing double mutants and performing growth assays on plates (Fig 6). We tested interactions with *tdp1Δ*, encoding the phosphodiesterase that removes Top1 adducts [59]; *mre11Δ*, encoding the nuclease required for resection at breaks [60]; and *mus81Δ*, an endonuclease required for fork reversal following CPT treatment [61]. Additionally, recent studies have implicated XPF nucleases in cleavage during CPT response in non-replicating cells [62] so we examined *rad16*Δ (part of the XPF nuclease; [63]). We also examined *rad2Δ*, which encodes the FEN-1 endonuclease [64]. Recent research in human cells showed FEN1 endonuclease is epistatic with XPF for CPT damage repair [41].

We observed a modest reduction in colony size on YES at 36°C for *mst1-W66R mus81Δ* or *mst1-W66R mre11Δ*, compared to the single mutants, indicating a synthetic phenotype even in the absence of exogenous genotoxic stress. The *mus81Δ* single mutant is extremely CPT sensitive even at 0.1μM CPT, and the double mutant was completely inviable on CPT. We observed that *mst1-W66R mre11Δ* and *mst1-W66R tdp1Δ* were more sensitive than either single mutant to low doses of CPT. These data suggest the role of *mst1-W66R* is separate from *mre11Δ*, *mus81Δ* or *tdp1Δ* since the combined effect is more dramatic than either parent.

The *rad16Δ* mutant shows minimal CPT sensitivity, while the double mutant *rad16Δ mst1-W66R* resembles *mst1-W66R*. While *rad2Δ* has modest CPT sensitivity by itself, again the double mutant resembles *mst1-W66R* alone. However, *rad16Δ rad2Δ* double mutant has increased CPT sensitivity relative to either single mutant, suggesting that they work in separate pathways.

### Epistatic interactions of Mst1 with XPF1 and FEN1 endonclease

Since the *mst1-W66R* CPT growth phenotype is epistatic to both *rad16Δ* and *rad2Δ*, we investigated how these mutants interact by examining recruitment of RPA, Rad52 and Rad54 to repair foci.

Untreated *rad16Δ* cells have more RPA foci than wild type, but this increase was partly rescued in the *mst1-W66R rad16Δ* double mutant (Fig 5A). However, following a four-hour CPT treatment, the percentage of cells with RPA foci increased to about 40% in both single and double *rad16Δ* mutants (S2 Table). Although this percentage was not as high as in wild type, it is significantly higher than the RPA foci observed in CPT-treated *mst1-W66R* cells.

Untreated *rad16Δ*, *mst1-W66R*, and wild type all showed similar percentages of Rad52 foci (Fig 5B). However, following CPT treatment, *rad16Δ* showed dramatic increase of cells with Rad52 compared to wild type. A similar high percentage was also observed in the *rad16Δ mst1-W66R* double mutant. Notably, contrasting with the Rad52 results, there was no increase in Rad54 foci upon CPT treatment in either *rad16Δ* and *rad16Δ mst1-W66R* (Fig 5C). Despite the absence of CPT sensitivity in *rad16Δ*, this mutation dramatically changes the recruitment of Rad52 and Rad54, in opposite directions.

We examined protein recruitment in *rad2Δ* single and double mutants. Similarly to *rad16Δ*, *rad2Δ* mutants had modestly increased cells with RPA foci than wild type without treatment (Fig 5A). *rad2Δ* also partially rescued the defects of recruiting RPA foci in *mst1-W66R* upon CPT treatment. However, like *mst1-W66R*, *rad2Δ* mutants showed very little increase in Rad52 protein recruitment following CPT treatment when comparing to untreated (Fig 5B and S2 Table). However, recruitment of Rad54 foci is unchanged in *rad2Δ* mutants compared to wild type (Fig 5C). Thus, *rad2*Δ has opposite effects on repair focus formation compared to *rad16Δ*.

Finally, we examined *rad2Δ rad16Δ*, which is strikingly more sensitive to CPT than either single mutant. The double mutant showed slightly more cells with RPA foci when treated with CPT (Fig 5A). Rad52 foci were notably lower in *rad2Δ rad16Δ* than the *rad16Δ* or *rad16Δ mst1-W66R* strains. (Fig 5B and 5C). Similarly, the accumulation of Rad54 foci in *rad2Δ rad16Δ* was intermediate between the levels observed for the two single mutants in CPT. The data suggest that Rad2 and Rad16 in *S*. *pombe* are not in a common pathway for CPT repair response.

## Discussion

The Kat5 acetyltransferase in *S*. *pombe* Mst1 is the catalytic subunit of the NuA4 histone acetyltransferase complex, and makes pleiotropic contributions to genome stability (rev. in [4, 63]). In our previous reports, we showed that Mst1 is essential for viability and facilitates acute DSB repair by promoting long-range resection that depends on Exo1 [14, 17]. We found that Mst1 binding to DSB requires Clr4, the H3K9 methyltransferase in *S*. *pombe* [17, 51, 52]. This is consistent with data from metazoans that indicate a role for H3K9^me^ in targeting Kat5 proteins to sites of DNA damage [16, 36, 37].

Curiously, the *S*. *cerevisiae* Kat5 orthologue Esa1 also contains a conserved chromodomain, despite the absence of H3K9^me^ and its associated heterochromatin in this organism [65]. The chromodomain mutation *esa1-W66R* creates sensitivity to various genotoxins, suggesting a role in repair response that is independent of H3K9^me^ [35]. We constructed the equivalent fission yeast *mst1-W66R* to examine H3K9-dependent and independent roles for this domain in fission yeast.

*S*. *pombe mst1-W66R* is not sensitive to MMS, HU, or DSB-inducing drug Bleomycin, and unexpectedly only showed sensitivity to CPT at 36°C (Fig 2). CPT leads to damage by stalling the topoisomerase I cleavage complex (Top1cc) on DNA (rev. in [39]), leading to S-phase dependent DNA breaks [66]. CPT also affects formation of DNA-RNA hybrids due to replication-transcription collision [41]. Our data show that *mst1-W66R* has defects in long-range resection but can still (albeit slowly) recruit recombination protein Rad52 to an induced DSB, suggesting a long-range resection independent but Rad52-dependent pathway is activated. We posit this might be due to single strand annealing or MMEJ, but this will require further examination.

In contrast to the hypomorph *mst1-L344S*, we observed only minimal changes in transcription in *mst1-W66R*, none of which obviously linked to the CPT repair response. Of 52 differentially expressed genes, only one of them overlaps with the differentially expressed genes in *mst1-L344S* [44]. This suggests that the *W66R* allele does not substantially affect general transcriptional regulation by Mst1. We also compared the transcriptome in CPT-treated *mst1-W66R* to that of the untreated one, and most of the differentially expressed genes were related to metabolism. We conclude that damage-induced transcription is not the major regulator of the CPT response in *mst1-W66R*.

Methylation of H3K9 by Clr4 and subsequent binding of chromodomain proteins Swi6/HP1 and Chp1 define the pericentromeric heterochromatin in *S*. *pombe* (rev. in [45]). Mst1 has previously been shown to contribute to recruitment of Swi6 by acetylating H3K4 [13]. We found that *mst1-W66R* has defects in silencing at outer repeats of centromere starting at 32°C and increased at 36°C. (Figs 2B and 4). Loss of silencing typically correlates to centromere dysfunction leading to disruptions in chromosome segregation and sensitivity to the microtubule poison TBZ [47, 67, 68], rev. in [46]) and we observed both of these phenotypes (Fig 2).

Assembly of the pericentromere heterochromatin via RNAi machinery is temperature sensitive [45, 48]. This likely reflects the mislocalization of the Dicer protein Dcr1 at higher temperature [54]. Although CPT is widely viewed as an S-phase specific inhibitor that blocks replication fork progression, it also affects resolution of transcription [69]. Interestingly, in addition to its role promoting heterochromatin assembly and H3K9 methylation, Dcr1 is also important to resolve DNA/RNA hybrids to reduce transcription/replication collisions [70]. We reasoned that increased transcription at the pericentromere due to temperature-dependent loss of silencing could create conditions that increase CPT sensitivity. Therefore, we hypothesized that centromere silencing defects and CPT-sensitivity phenotypes in *mst1-W66R* could be linked, and mutants affecting heterochromatin formation might also have growth defects in CPT. If this were the case, then mutations that eliminate heterochromatin formation at the pericentromere would increase CPT sensitivity. This was not what we observed: *clr4Δ*, the mutant of H3K9 methyltransferase [51, 52]; *swi6Δ*, the mutant of HP1 [51]; as well as *dcr1Δ*, the mutant of Dicer protein [53] did not affect CPT sensitivity at lower temperatures. However, all these mutants showed CPT sensitivity at 36°C. Therefore, this CPT sensitivity is not due to increased transcription in the centromere (which occurs at all temperatures in heterochromatin mutants). This suggests that proteins important for heterochromatin establishment at centromere are also required for the proper response in CPT-induced damage repair. Importantly, we observed that heterochromatin mutants appeared less sensitive to CPT than *mst1-W66R*, while double mutants with *mst1-W66R* showed increased sensitivity to CPT relative to either single mutant. We conclude that Mst1’s response to CPT-induced damage response is at least partially independent from heterochromatin formation and H3K9^me^ binding.

One possibility is that the *mst1-W66R* mutant is temperature sensitive for some of its activities, although it is clearly competent to fulfill its essential functions. However, we observe that sensitivity of *mst1-W66R* to CPT is observed even in the presence of wild type Mst1, indicating a dominant negative effect. This may be due to a conflict between wild type and mutant forms of the NuA4 complex, where the mutant complex is recruited to some sites and inhibits appropriate interactions and argues against a simple temperature sensitive defect.

We examined whether CPT treatment impacts silencing directly. Consistent with previous studies that RNAi machinery is partially impaired at restrictive temperature [47, 48], we observed reduced silencing of a reporter at 36°C compared to 32°C. CPT treatment modestly reduced expression at outer repeats at 32°C. We reasoned that such de-silencing could result from -transcription collisions at the centromere, and thus inhibition of siRNA production to silence the centromere. However, expression was not further increased at 36°C or in the *W66R* background, suggesting CPT-induced de-silencing is independent of RNAi machinery and Mst1. Finally, CPT treatment in *swi6Δ* led to higher expression at outer repeats compared to the untreated, suggesting the CPT-induced de-silencing is also independent of heterochromatin assembly. These data suggest that CPT interferes with silencing in some other way.

The increased sensitivity of *mst1-W66R* in CPT-induced de-silencing suggests it has a role in repair of CPT-induced damage, apart from its silencing function at centromere. Indeed, we found that *mst1-W66R* disrupts recruitment of repair proteins RPA, the ssDNA binding protein; recombination protein Rad52 (Fig 5), suggesting the chromodomain of Mst1 might regulate resection at CPT-induced damage, and facilitate Rad52-mediated repair pathways. In contrast, Mst1 does not affect the recruitment of homologous recombination protein Rad54 following CPT treatment. The presence of multiple dispersed repair foci in one nucleus in *mst1-W66R* upon CPT treatment suggests that the genome-wide damage generated by CPT treatment is not limited to the centromere domain (S4B Fig). The formation of ssDNA can be driven by a variety of pathways including resection, recombination, and/or helicase uncoupling [71]. The reduction in ssDNA signal in *mst1-W66R* therefore could reflect any of these pathways but would be consistent with the reduced resection we observe in the DSB repair assay.

We constructed candidate double mutants to assess what pathways may be affected by *mst1-W66R* and assessed CPT sensitivity in chronic exposure. We find that *mst1-W66R* is epistatic to *rad16Δ* the homolog of XPF endonuclease that cleaves the 3’end of DNA-protein adducts [41, 62]. Repair of CPT damage is complex because the drug blocks Top1 in the cleavable complex and produces DSBs which are repaired during S-phase. The growth data in Fig 6A shows that the growth of the *rad16Δ mst1-W66R* double mutant is worse than *rad16Δ* single and comparative to *mst1-W66R*, though not identical, suggesting that *rad16*^*+*^ has functions independent of *mst1*^*+*^. However, Fig 5C shows that the *mst1-W66R* epistatic phenotype to *rad16Δ* does not extend to recruitment of repair factors to the DSB (e.g. *rad16Δ* shows the same phenotype as *mst1-W66R rad16Δ)*. Thus, the growth defects observed in Fig 6 represent Mst1-Rad16 interactions not related to recruitment of repair factors. However, the interactions between *mst1-W66R* and *rad2Δ*, the FEN-1 endonuclease known for its function in Okazaki fragment processing, do extend to recruitment of repair factors as the double mutant does have different recruitment defects than the single mutants (Fig 5), In other systems, Fen1 was shown to work in an epistasis group with XPF in CPT damage response [41, 64]. Therefore, not unexpectedly, in fission yeast *rad16Δ rad2Δ* showed increased CPT sensitivity.

We observed that both *rad16Δ* and *rad2Δ* mutants showed accumulation of RPA foci when untreated, suggesting the presence of endogenous damage. RPA foci were further induced following CPT treatment. Although *mst1-W66R* alone does not induce RPA signal, the double mutant with *rad16* shows significant increase (like the *rad16* single mutant), while the double mutant with *rad2* shows no induction. However, these mutants also diverged in their recruitment of Rad52. The *rad2Δ* cells had increased Rad52 foci in both as single and double (*mst1-W66R)* mutants when untreated, but it did not increase upon CPT treatment. In contrast, *rad16Δ* did not show accumulation of Rad52 foci when left untreated, but significantly increased the percentage after CPT treatment. We hypothesize that Mst1 repairs the genome-wide CPT-induced damage through promoting FEN1 and inhibiting XPF endonucleases. This suggests that unlike human cells, XPF endonuclease inhibits the Rad52-mediated SSA repair at CPT-induced damage in *S*. *pombe*, but promotes Rad54-mediated fork reversal [72]. FEN1 also does not work in the same pathway with XPF to remove R-loops induced by CPT as in human cells [41], but it facilitates the Rad52-mediated SSA while it has little impact on fork reversal.

It is interesting that the *esa1-W66R* mutant in budding yeast leads to a similar CPT phenotype despite the absence of H3K9^me^ in that organism [35]. Similarly, we find that fission yeast *mst1-W66R* does not phenocopy *clr4Δ* especially upon CPT treatment. One possibility is that other methylations might recruit the Mst1 chromodomain in response to CPT. There is evidence that mammalian Kat5 chromodomain has affinity to H3K4me, H3K27me, H3K36me, and H4K20me, in addition to H3K9^me^ [73]. In human cells, H3K36^me^ by SETD2 results in survival defects in CPT [74]. In *S*. *pombe*, cells without H4K20^me^ also show higher sensitivity to CPT. Some studies suggest that the *S*. *cerevisiae* Esa1 chromodomain may target unmodified histones, or RNA [75, 76]. Additionally, some evidence in *S*. *pombe* suggests that chromodomain protein binding to RNA antagonizes recognition of methylated histone [77, 78]. It is possible that the Mst1 effect on heterochromatin silencing or CPT response is mediated by an RNA binding function of the chromodomain.

## Materials and methods

### Strains and media

Fission yeast cells were grown in YES (yeast extract with supplements) or PMG (pombe minimal glutamate) with appropriate supplements [79]. Yeast strains used in this research are listed in S1 Table in the supplemental material.

### Construction of *mst1-W66R*

*mst1-W66R* was made by site-directed mutagenesis using Phusion Site-Directed Mutagenesis Kit with primers 5’Phos/CGTTTAGATGAAAGGATTACAATAGAT (Forward) and 5’Phos/TTTATTGTAGCATTATAGTG (Reverse). The *mst1-W66R* sequence was then cloned into pEBG78 (pTZ*ura4*^*+*^ 5’/3’UTR *mst1* (construct to disrupt *mst1*^*+*^ with *ura4*^*+*^). *mst1-W66R* construct was then PCR amplified and transformed into genome.

### Serial dilution plating

Yeast cell cultures were grown at 32°C in YES for one day. Cultures were diluted in YES to equal concentrations. Five-fold serial dilutions of the cultures were then spotted onto YES plates containing different concentrations of drugs. Plates were incubated at 32°C or 36°C for four days before scanning.

### Silencing assay

Silencing assays were performed as described previously [80] with the following modifications. Cells were grown to mid-log phase to 5X10^6^ cells/ml and spotted on selective medium with 1:5 serial dilutions. For *ura4*^*+*^ expression, cells were spotted on YES or non-selective with 1 g/L of 5’ FOA. Cells were grown at 32°C or 36°C for 4 days.

### RT-PCR

RT-PCR was performed as described previously [50]. Cells were grown to mid-log phase. Total RNA was extracted using Qiagen RNeasy isolation kit according to manufacturer’s instructions and treated with TURBO DNA-free kit following routine DNase treatment instructions. Total RNA was resuspended in TE and checked for integrity by agarose electrophoresis. The cDNA strand was synthesized with 1μg RNA using Invitrogen SuperScript First-strand cDNA synthesis kit following the manufacturer’s instructions. The amount of *ura4*^*+*^ RNA expression was determined by PCR and analyzed by Amersham Typhoon laser and Fiji ImageJ. Three biological replicates were assessed for consistency. Results were plotted as fold expression relative to the mini-gene *ura4*-DS/E, located at the endogenous *ura4*^*+*^ locus.

### Live-cell imaging and quantitative measurements

Live cells imaging was performed as described in [81]. Yeast cell cultures were grown at 32°C in YES overnight. Cells were transferred into PMG + HULAA (Histidine, Uracil, Leucine, Adenine, Arginine) liquid cultures at 36°C for 16 hours before treating with 5μg/mL camptothecin for four hours. Cells were collected at OD_595_ 0.3–0.6, concentrated by centrifuging 1ml at 1500g for 1min and resuspended in 40μl. Concentrated cells were placed on a thin-film pad of 2% agarose in PMG+HULAA on a glass slide. A coverslip was added. Live cells were imaged on DeltaVision Core microscope with softWoRx v4.1 (GE, Issaquah, WA), using a 60X lens, and then deconvolved and projected in softWoRx software. Images were acquired in 13 0.2μm z-sections, then deconvolved and Maximum Intensity Projected (softWoRx, default settings). Two separate fields were imaged in each experiment, and 2 to 4 biological replicates were assessed for consistency. Images for publication were contrast adjusted using an equivalent histogram stretch on all samples. Color balance was adjusted, and scale bars were added in Fiji [82]. Significance was calculated using the Mann-Whitney U test.

### LacO LacI-mCherry DSB array colocalization with CFP-tagged Rad52

The assay was performed as described in [42]. In this system, the HO endonuclease is driven by the nmt1 promoter, which takes approximately 20 hours to induce following removal of thiamine from the media [83]. The assay can quantitively monitor resection and DSB repair factors recruitment to the break. A difference in fluorescent signal between WT controls and mutants demonstrates a resection and recruitment failure. Cells were cultured at 36°C in PMG + HULAA + Thiamine liquid media to OD595 of 0.4–0.6. Cells were then washed twice with PMG + HULAA medium and incubated at 36°C for 19 hours to induce the HO-driven DSB break. Following induction, cells were collected at OD595 of 0.3–0.6, and were processed and imaged as described above.

### mRNA sequencing and gene expression analysis

Yeast cell cultures were grown at 36°C for one day before treating with 15μM camptothecin for four hours. RNA was isolated from yeast cell culture using the Qiagen RNeasy kit according to manufactures instructions. 200 ng of RNA was used for gene expression analysis with NovaSeq PE150. Sequence reads quality was checked using FastQC 0.11.7. Sequence reads were then aligned using STARalign 2.7.0e and assembled using Cufflinks 2.2.1.

The counts were normalized using housekeeping genes (act1, adh1, atb2 and gad8) as control with methods in “RUVseq” package in R [84]. Downstream filtering, normalization, dispersion and model fitting, as well as differential expression were performed with “edgeR” package in R [85]. Multidimensional analysis and principal component analysis were performed using normalized counts-per-million generated using “cpm” function in “edgeR” to visualize sample variation, as well as to identify potential sample outliers and gene outliers respectively. Over-expression and gene set enrichment analysis were performed using R package “clusterProfiler” [86]. p-value cut-off was set to 0.05 for GO over-representation test and Kyoto Encyclopedia of Genes and Genomes (KEGG) over-representation test, 0.3 for GO gene set enrichment analysis (GSEA). Gene list for GSEA were prepared based on the order of log2 (Fold change).

## Supporting information

S1 Fig The conserved chromodomain sequences. Chromodomain sequences of the indicated coordinates were aligned using Clustal Omega-Multiple Sequence Alignment. All sequences are for strain 972h- from PomBase.
(TIF)

S2 Fig *mst1-W66R* mutant characterization in transcriptome. (A)-(B) Volcano plot of differentially expressed genes (p-value cutoff = 0.05, log2(Fold change) cutoff = 1.5) in (A) untreated *mst1-W66R* compared to untreated wild type. (B) CPT treated *mst1-W66R* compared to untreated *mst1-W66R*. (C)-(D) KEGG analysis of (C) upregulated and (D) downregulated pathways in CPT treated *mst1-W66R* compared to untreated *mst1-W66R*.
(TIF)

S3 Fig *mst1-W66R* mutants sensitivity to CPT are temperature specific and *mst1-W66R* is a dominant negative allele. (A) *clr4Δ*, *swi6Δ*, *dcr1Δ* and the double mutants with *mst1-W66R* showed sensitivity to CPT at 32°C. (B) *mst1-W66R* cells complemented with plasmid expressing wild type Mst1 under native promoter. Cells were grown in YES media overnight at 32°C then 5X serial dilutions were spotted onto YES plates or YES plates containing CPT. Plates were incubated at 32°C or 36°C and photographed after 4 days.
(TIF)

S4 Fig *mst1-W66R* affects the repair of CPT-induced damages genome-wide. **Top:** Percentage of cells with single focus or multi-foci of (A) RPA (Rad11) (B) Rad52 and (C) Rad54 foci in WT, *mst1-W66R*, *rad2Δ*, *rad2Δ mst1-W66R*, *rad16Δ*, *rad16Δ mst1-W66R* and *rad2Δ rad16Δ*. **Bottom:** Examples of cells with multi-foci after CPT treatment. Rad11-CFP is colored magenta for visibility.
(TIF)

S1 Raw images Raw images of Fig 4. (PDF)

S1 Table Strains used in this study. (XLSX)

S2 Table Differential-expressed genes from mRNA-sequencing. (XLSX)

S3 Table Average ± standard error of cells with Rad11, Rad52 or Rad54 shown in Fig 5. (XLSX)
