# Correction: Chromodomain mutation in *S*. *pombe* Kat5/Mst1 affects centromere dynamics and DNA repair

**DOI:** 10.1371/journal.pone.0361647

**Published:** 2026-10-27

**Authors:** Tingting Li, Ruben C. Petreaca, Joshua M. Park, Susan L. Forsburg

Joshua M. Park should be included in the author byline. Joshua M. Park should be listed as the third author and affiliated with Molecular & Computational Biology, University of Southern California. The contributions of this author are as follows: Formal Analysis, Investigation, Methodology, Resources, Validation, Visualization and Writing – Review & Editing.

The correct citation is: Li T, Petreaca RC, Park JM, Forsburg SL (2024) Chromodomain mutation in *S*. *pombe* Kat5/Mst1 affects centromere dynamics and DNA repair. PLoS ONE 19(4): e0300732. https://doi.org/10.1371/journal.pone.0300732
