# Supplementary figures and images for: Chromodomain mutation in S. pombe Kat5/Mst1 affects centromere dynamics and DNA repair
Source: PLoS One. 2024 Apr 25;19(4):e0300732. doi: 10.1371/journal.pone.0300732 (PMC11045136; doi:10.1371/journal.pone.0300732)

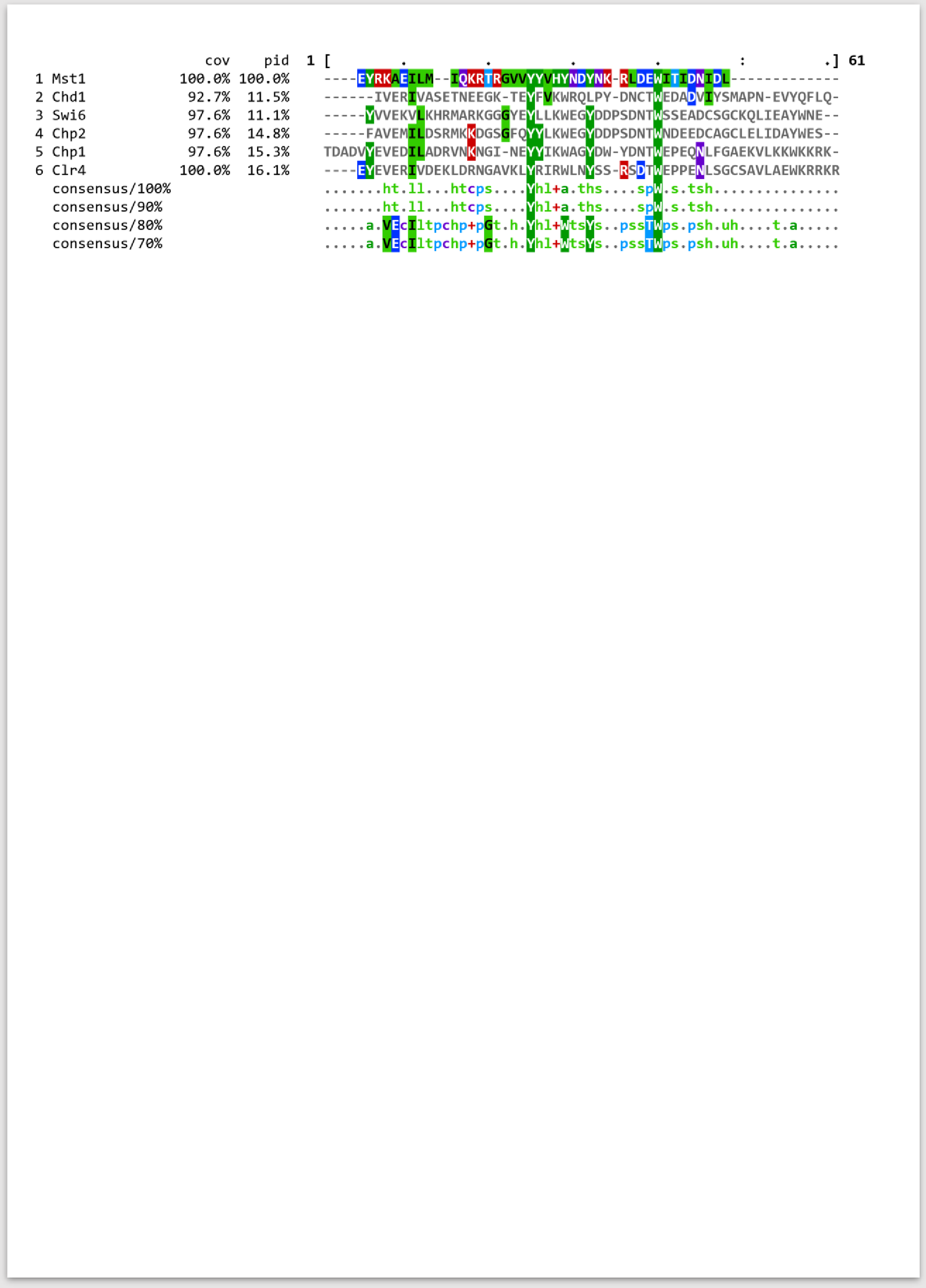

Supplement: S1 Fig — Chromodomain sequences of the indicated coordinates were aligned using Clustal Omega-Multiple Sequence Alignment. All sequences are for strain 972h- from PomBase. (TIF) [file pone.0300732.s001.tif]

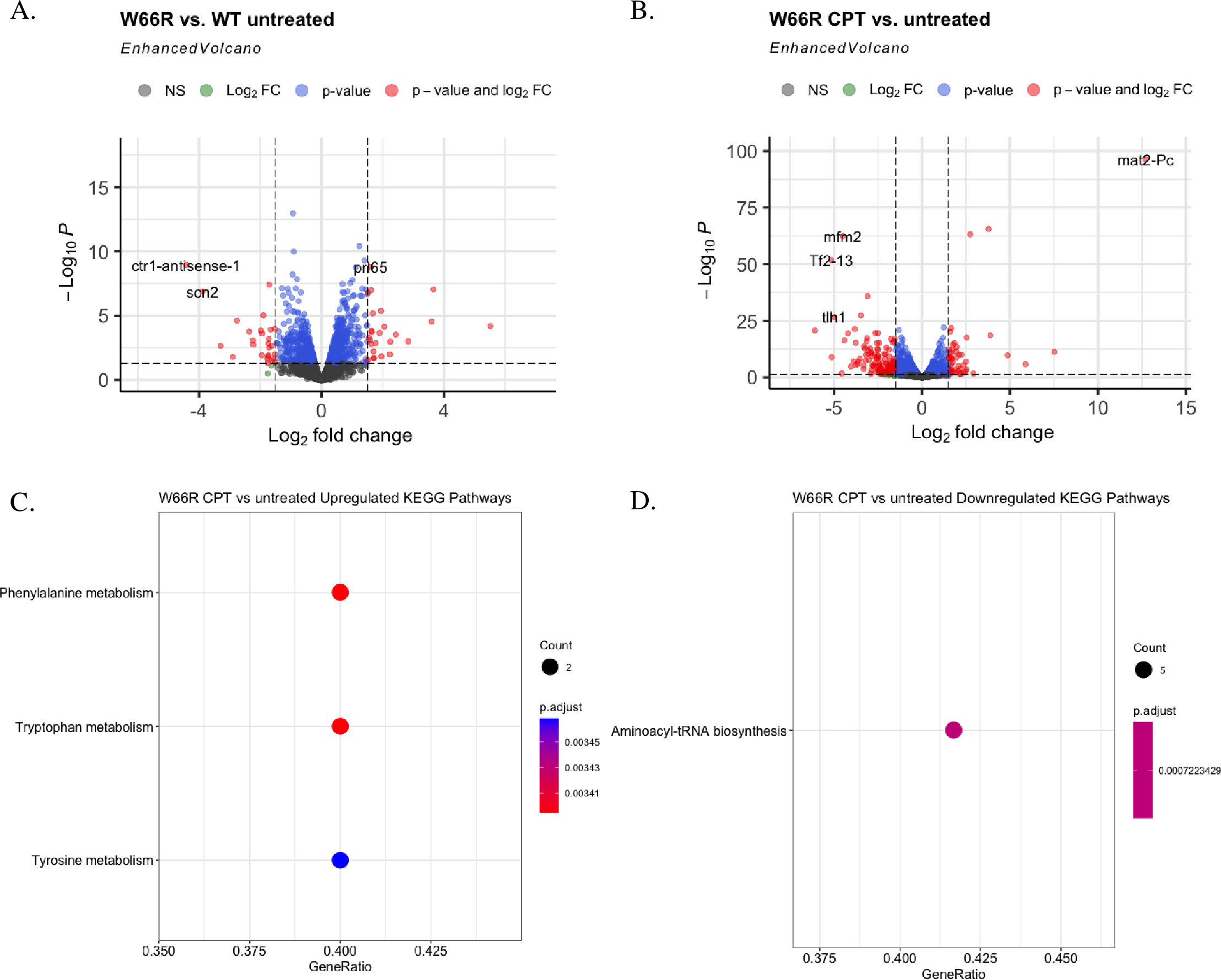

Supplement: S2 Fig — (A)-(B) Volcano plot of differentially expressed genes (p-value cutoff = 0.05, log2(Fold change) cutoff = 1.5) in (A) untreated mst1-W66R compared to untreated wild type. (B) CPT treated mst1-W66R compared to untreated mst1-W66R. (C)-(D) KEGG analysis of (C) upregulated and (D) downregulated pathways in CPT treated mst1-W66R compared to untreated mst1-W66R. (TIF) [file pone.0300732.s002.tif]

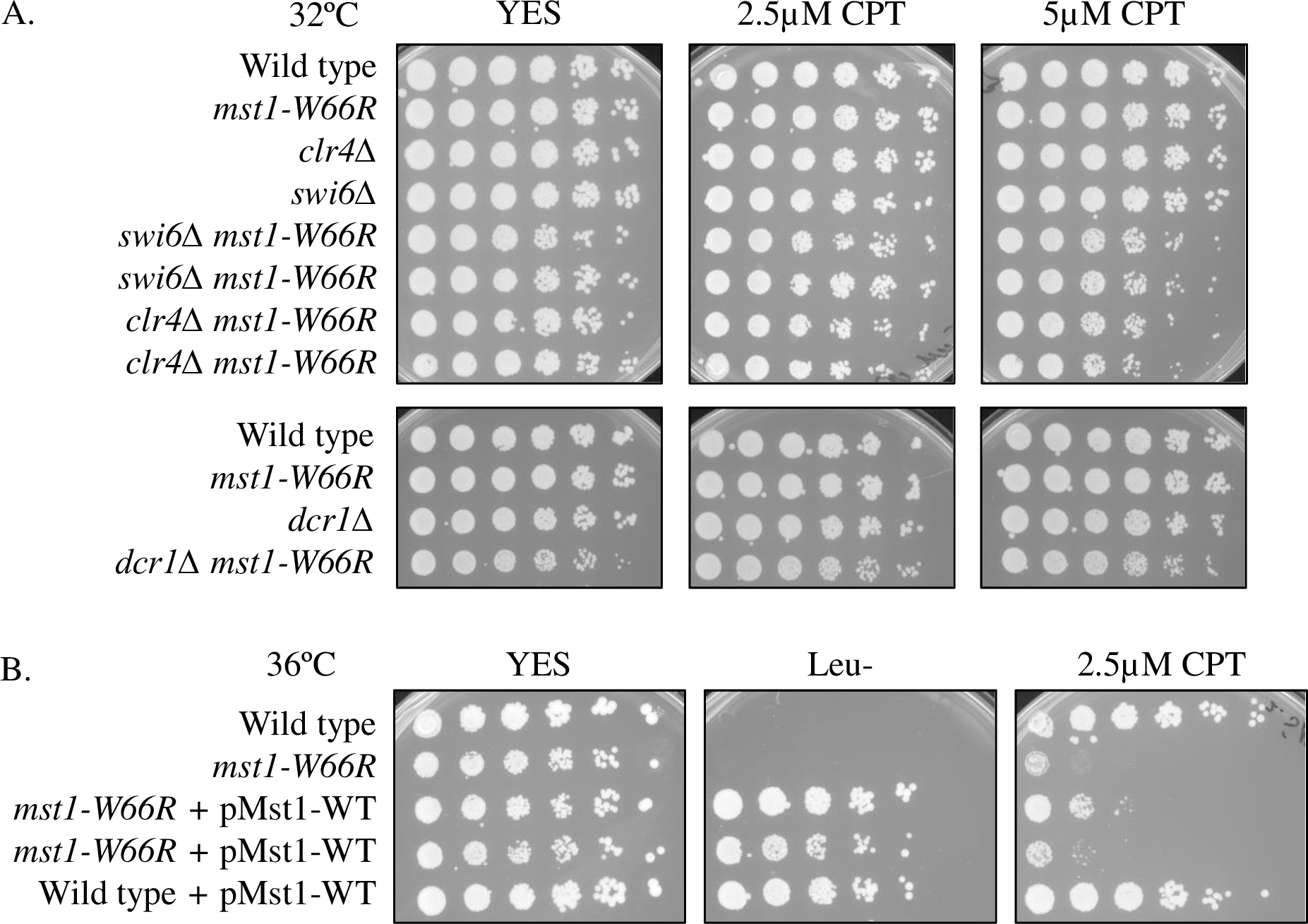

Supplement: S3 Fig — (A) clr4Δ, swi6Δ, dcr1Δ and the double mutants with mst1-W66R showed sensitivity to CPT at 32°C. (B) mst1-W66R cells complemented with plasmid expressing wild type Mst1 under native promoter. Cells were grown in YES media overnight at 32°C then 5X serial dilutions were spotted onto YES plates or YES plates containing CPT. Plates were incubated at 32°C or 36°C and photographed after 4 days. (TIF) [file pone.0300732.s003.tif]

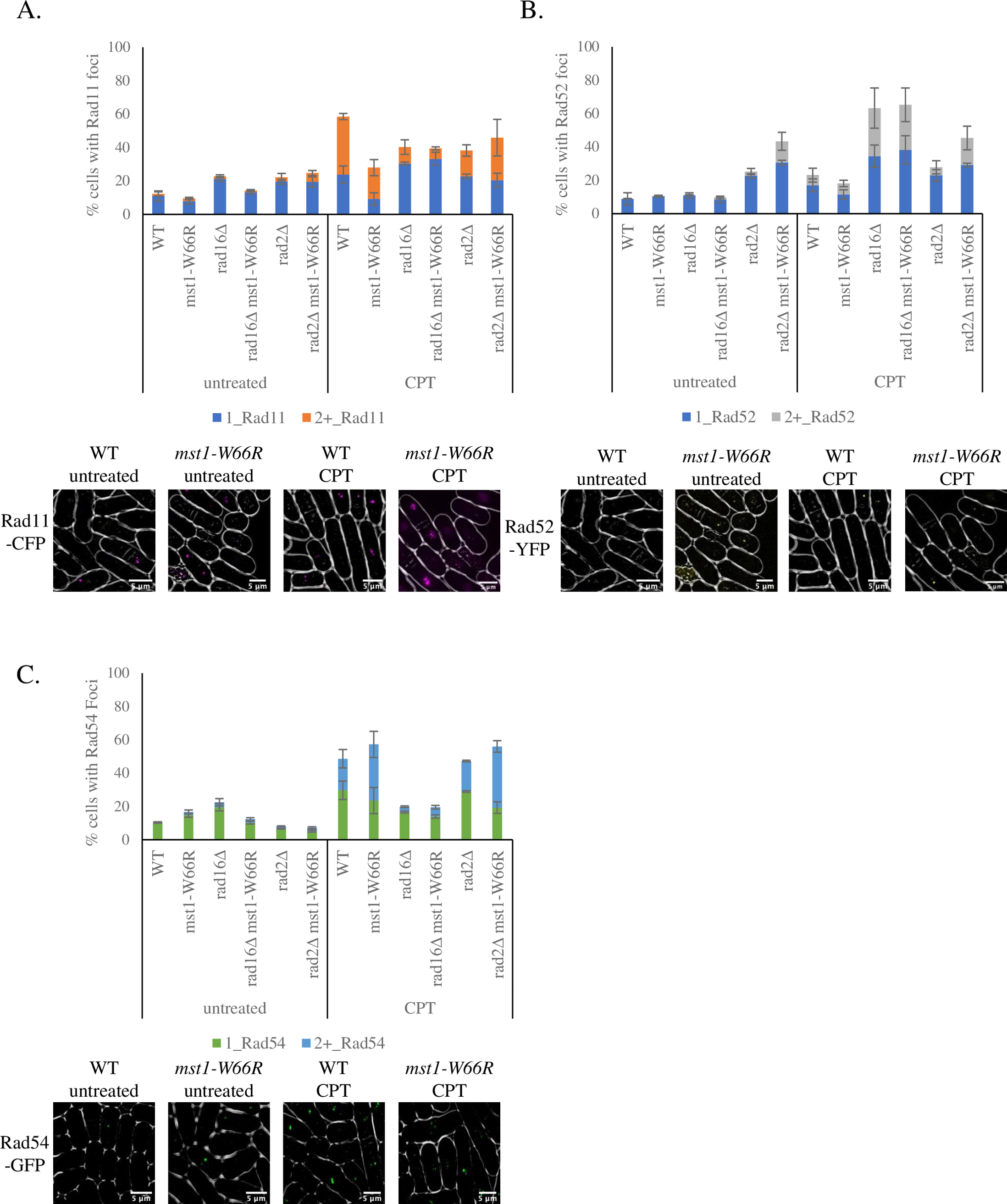

Supplement: S4 Fig — Top: Percentage of cells with single focus or multi-foci of (A) RPA (Rad11) (B) Rad52 and (C) Rad54 foci in WT, mst1-W66R, rad2Δ, rad2Δ mst1-W66R, rad16Δ, rad16Δ mst1-W66R and rad2Δ rad16Δ. Bottom: Examples of cells with multi-foci after CPT treatment. Rad11-CFP is colored magenta for visibility. (TIF) [file pone.0300732.s004.tif]
