# Supplementary material for: Chromodomain mutation in S. pombe Kat5/Mst1 affects centromere dynamics and DNA repair
Source: PLoS One. 2024 Apr 25;19(4):e0300732. doi: 10.1371/journal.pone.0300732 (PMC11045136; doi:10.1371/journal.pone.0300732)

Figure 4 RT-PCR Raw Image:

A) Lane 1: wild type 32° C; Lane 2: wild type 32° C following CPT treatment
B) Lane 3: wild type 36° C; Lane 4: wild type 36° C following CPT treatment; Lane 5: *mst1-W66R* 36° C; Lane 6: *mst1-W66R* 36° C following CPT treatment
C) Lane 5: *swi6*Δ 36° C; Lane 6: *swi6*Δ 36° C following CPT treatment; Lane 7: wild type DNA

A).

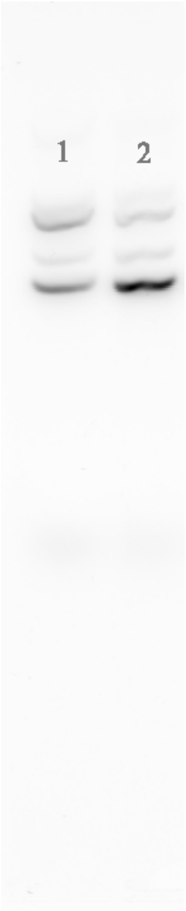

B).

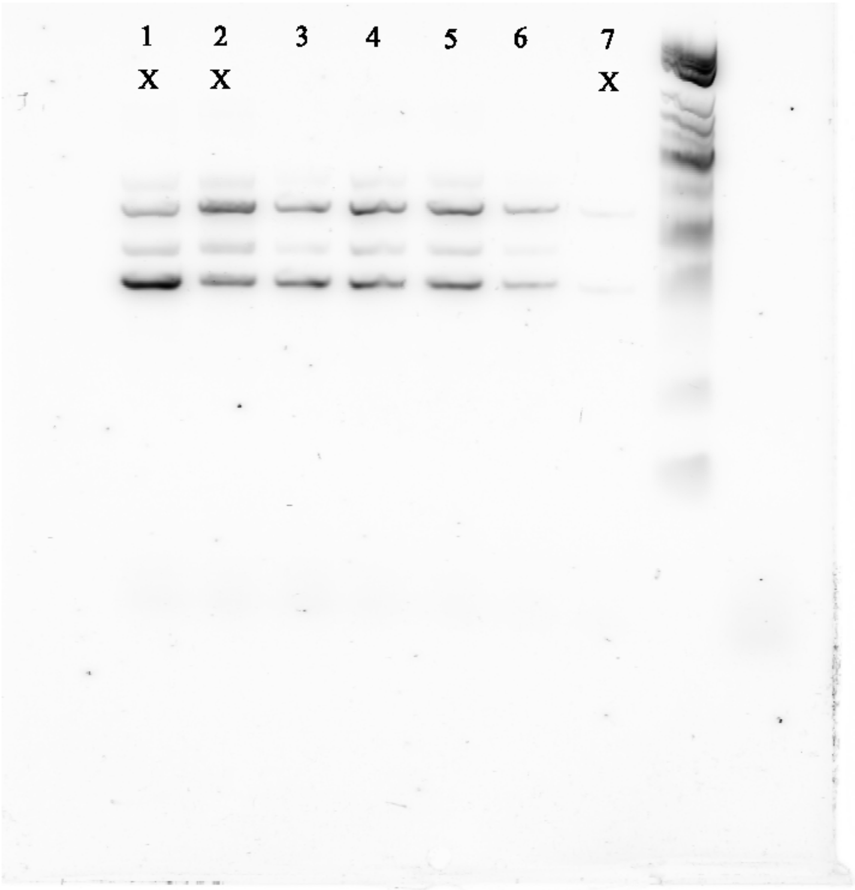

C).

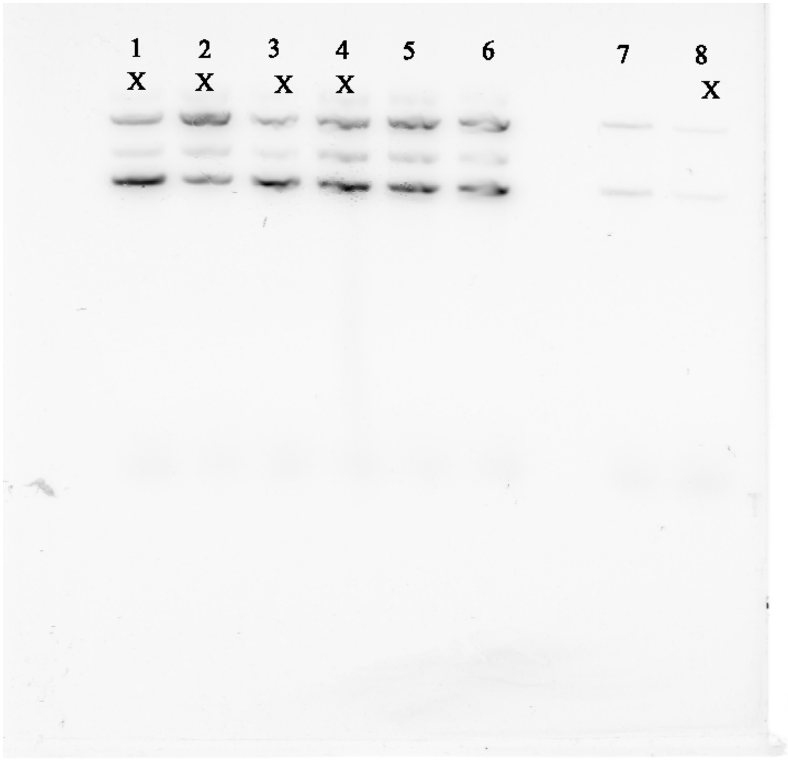

Supplement: S1 Raw images — (PDF) [file pone.0300732.s005.pdf]
